# miR-125a-5p attenuates macrophage-mediated vascular dysfunction by targeting Ninjurin1

**DOI:** 10.1038/s41418-021-00911-y

**Published:** 2022-01-01

**Authors:** Su Jung Hwang, Bum Ju Ahn, Min-Wook Shin, Ye-Seul Song, Youngbin Choi, Goo Taeg Oh, Kyu-Won Kim, Hyo-Jong Lee

**Affiliations:** 1 School of Pharmacy, Sungkyunkwan University, 2066 Seobu-ro, Jangan-gu, Suwon, Gyeonggi-do 16419 South Korea; 2 College of Pharmacy, Inje University, 607 Obang-dong, Gimhae, Gyungnam 621-749 South Korea; 3 College of Pharmacy and Research Institute of Pharmaceutical Sciences, Seoul National University, Seoul, 08826 South Korea; 4 Immune and Vascular Cell Network Research Center, National Creative Initiatives, Department of Life Sciences, Ewha Womans University, Seoul, 03760 South Korea

**Keywords:** Extracellular matrix, Metabolic disorders, Chronic inflammation

## Abstract

Ninjurin1 (Ninj1), an adhesion molecule, regulates macrophage function in hyaloid regression, multiple sclerosis, and atherosclerosis. However, its biological relevance and the mechanism underlying its function in vascular network integrity have not been studied. In this study, we investigated the role of Ninj1 in physiological (postnatal vessel formation) and pathological (endotoxin-mediated inflammation and diabetes) conditions and developed a strategy to regulate Ninj1 using specific micro (mi)RNAs under pathological conditions. Ninj1-deficient mice exhibited decreased hyaloid regression, tip cell formation, retinal vascularized area, recruitment of macrophages, and endothelial apoptosis during postnatal development, resulting in delayed formation of the vascular network. Five putative miRNAs targeting Ninj1 were selected using the miRanda algorithm and comparison of expression patterns. Among them, miR-125a-5p showed a profound inhibitory effect on Ninj1 expression, and miR-125a-5p mimic suppressed the cell-to-cell and cell-to-matrix adhesion of macrophages and expression of pro-inflammatory factors mediated by Ninj1. Furthermore, miR-125a-5p mimic inhibited the recruitment of macrophages into inflamed retinas in endotoxin-induced inflammation and streptozotocin-induced diabetes in vivo. In particular, miR-125a-5p mimic significantly attenuated vascular leakage in diabetic retinopathy. Taken together, these findings suggest that Ninj1 plays a pivotal role in macrophage-mediated vascular integrity and that miR-125a-5p acts as a novel regulator of Ninj1 in the management of inflammatory diseases and diabetic retinopathy.

## Introduction

Nerve injury-induced protein 1 (Ninjurin1, Ninj1) is induced by nerve injury and acts as an adhesion molecule [1]. Ninj1 protein has two transmembrane domains and an adhesion motif in extracellular N-terminal (ENT) domains [2]. During development, Ninj1 expression is upregulated in leukocytes, facilitating hyaloid vessels atrophy through apoptosis of vascular endothelial cells (VECs) [3]. Additionally, Ninj1 mediates macrophages recruitment in multiple sclerosis (MS) [4]. In an experimental autoimmune encephalomyelitis model, Ninj1 promoted protrusive membrane dynamics, transendothelial migration, and basal motility of leukocytes [5]. Therefore, inflammatory stimuli induce Ninj1 expression in leukocytes, enhancing adhesion to VECs and the extracellular matrix (ECM) and recruitment to the site of inflammation [6]. Various trials have been conducted to regulate the inflammation mediated by Ninj1-neutralizing antibodies, small interfering RNA (siRNA), peptides, and natural products [4, 7–9]. However, a strategy to effectively regulate Ninj1 expression has not yet been established.

Angiogenesis is the formation of new blood vessels from pre-existing vasculatures related to wound healing and ischemia [10–12]. Retinal angiogenesis results from abnormal activation of blood vessels, leading to retinopathy of prematurity (ROP), diabetic retinopathy (DR), and age-related macular degeneration (AMD), resulting in vision loss [13–15]. Under pathological conditions, hypoxia stabilizes hypoxia-inducible factor-1 (HIF-1), which stimulates the expression of proangiogenic target genes, such as vascular endothelial growth factor (VEGF) [16]. Therefore, therapeutic interventions against vascular diseases have focused on angiogenic factors, and anti-VEGF therapy is the standard therapeutic regimen in ophthalmology [17–19]. However, VEGF blockade is ineffective in some patients, and systemic adverse effects have also been reported [20]. For example, bevacizumab, an anti-VEGF antibody, can cause perforation and proteinuria [21]. Consequently, there is considerable demand for new drugs that efficiently stabilize pathological blood vessels without adverse effects.

Retinal vasculature is a suitable model for studying vascular biology because it is easy to observe and apply drugs locally, and it does not present problems related to first-pass metabolism and the blood–retinal barrier [22–24]. Because the retinal vascular network is composed of various types of cells and their interaction is crucial for homeostasis, cell adhesion proteins, such as Ninj1, could act as key targets for new therapeutic agents. Previously, we reported that Ninj1 induced leukocyte-mediated hyaloid atrophy, and found that Ninj1 neutralized hyaloid remodeling [3]. However, it is largely unknown whether Ninj1 regulates the development of retinal vessels, how Ninj1 functions in pathological conditions, such as DR, and how Ninj1 expression can be modulated.

In this study, we used two types of retinal models (postnatal retinal angiogenesis and DR) to investigate the role of Ninj1 and its underlying mechanism of action and establish a strategy to regulate Ninj1 expression. Postnatal angiogenesis is useful for screening for vascular defects [25]. Hyaloid vessels nourish the lens and retina with oxygen and nutrients during early development. After birth, retinal blood vessels begin to grow from the optic nerve head and cover the surface of the retina by postnatal day 8 (P8). Meanwhile, the hyaloids gradually undergo atrophy after birth and no longer exist by P21 [26]. Therefore, we observed the changes in retinal vascular network depending on Ninj1 ablation. We also used a streptozotocin (STZ)-induced DR model, which reflected clinical characteristics, such as leukocyte recruitment and vascular leakage [27]. Here, we investigated the role of Ninj1 in leukocyte recruitment and vascular leakage in STZ-induced DR. Based on these observations, we speculated that Ninj1, expressed by macrophages and blood vessels, may offer novel targets for therapeutic interventions in DR and AMD.

## Results

### Ninj1 mediated retinal angiogenesis during postnatal development

**Fig. 1:**
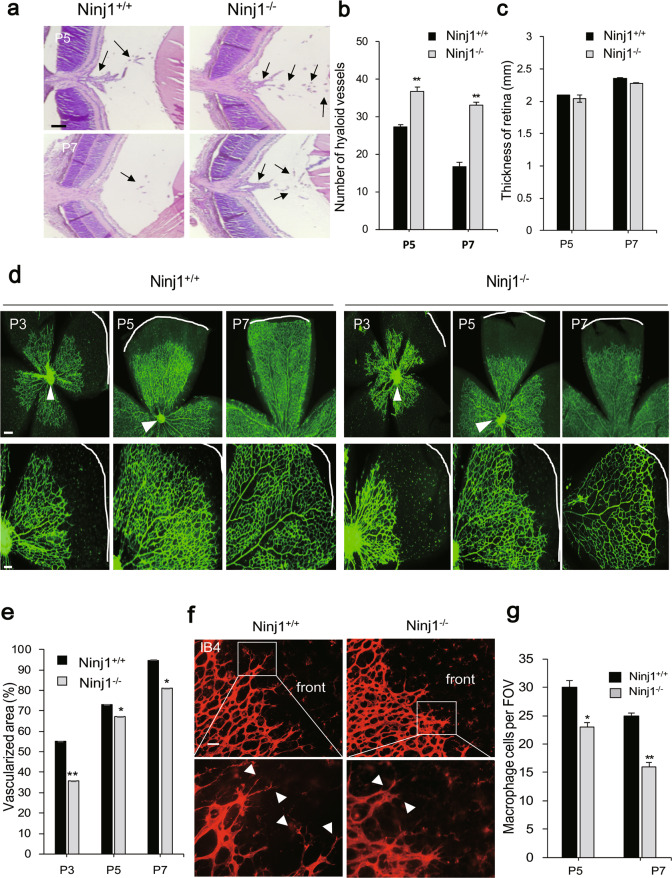
Ninj1 deficiency impaired postnatal retinal angiogenesis. **a** Hematoxylin and eosin (H&E) staining of Ninj1^+/+^ and Ninj1^−/−^ mice at P5 and P7. Black arrows represent the remaining hyaloid vessels. Scale bars, 0.1 mm. **b** Quantification of hyaloid vessel number for Ninj1^+/+^ and Ninj1^−/−^ mice. The number of hyaloid vessels was quantified from three independent eyes. Each bar represents mean ± S.D. (*n* = 8); ^**^P < 0.01, Ninj1^+/+^ vs. Ninj1^−/−^ mice. **c** Quantifi**c**ation of thickness of retina. Bar graph indicating total thickness of retina between wild-type and Ninj1^−/−^ mice. Each bar represents mean ± S.D. (*n* = 8); ^*^P < 0.05, wild-type vs. Ninj1^−/−^ mice. **d** Flat-mounted retina stained with isolectin B4 (green) of P3, P5, and P7 pups. Scale bar, 200 μm. **e** Quantification of vascularized area in retina. Bar graph indicates the ratio of vascularized area per retina from five mice per group in wild-type and Ninj1^−/−^ mice. Experiments were independently performed 3 times. ^*^P < 0.05, ^**^P < 0.01, wild-type vs. Ninj1^−/−^ mice. **f** Flat-mounted retina stained with isolectin B4 (red) at P5. The white arrowhead indicates the filopodia of tip cells. Scale bars, 50 μm. Experiments were independently performed 3 times. **g** Bar graph indicates the number of F4/80-positive cells per field of view (FOV). At P5 and P7, mouse eyes were immunostained with F4/80 in wild-type and Ninj1^−/−^ mice. Each bar represents mean ± S.D. (*n* = 5); ^*^P < 0.05, ^**^P < 0.01, wild-type vs. Ninj1^−/−^ mice.

We examined whether Ninj1 regulates retinal vascular development in Ninj1-deficient (Ninj1^−/−^) mice. Ninj1^−/−^ mice showed a greater number of hyaloids in cross-sectioned tissues than wild-type mice at P5 and P7 (Fig. 1a). The number of hyaloids decreased by 39.0% in the wild-type and by 10% in Ninj1^−/−^ mice from P5 to P7 (Fig. 1b). However, there was no significant difference in retinal thickness between wild-type and Ninj1^−/−^ mice (Fig. 1c). These data suggest that Ninj1 deficiency notably delayed the hyaloid regression. Next, we investigated changes in retinal vascular patterning depending on Ninj1 silencing. In the wild type, retinal vasculature began to grow at P3, approached the retina edge by P5, and was completely formed by P7 (Fig. 1d). However, Ninj1-deficient mice were not sufficiently covered with blood vessels even at P7 and had a less vascularized area (%) than wild-type mice, indicating delayed vascular development (Fig. 1e). Isolectin B4-staining (red) revealed sprouting and extension of filopodia at the leading edge of the growing vascular network in wild-type mice, but this phenomenon was inhibited in Ninj1-deficient mice (Fig. 1f). Next, we determined whether Ninj1 deficiency affected the recruitment of macrophages that interacted with retinal ECs (RECs) during vascular remodeling and guided the sprouting and fusion of tip cells [28]. The number of infiltrated macrophages in Ninj1-deficient mice was lower than that in wild-type mice at both P5 and P7 (Fig. 1g). Overall, these results suggest that Ninj1 plays an important role in retinal angiogenesis during postnatal development.

### Ninj1-expressing macrophages mediated apoptosis of RECs

**Fig. 2:**
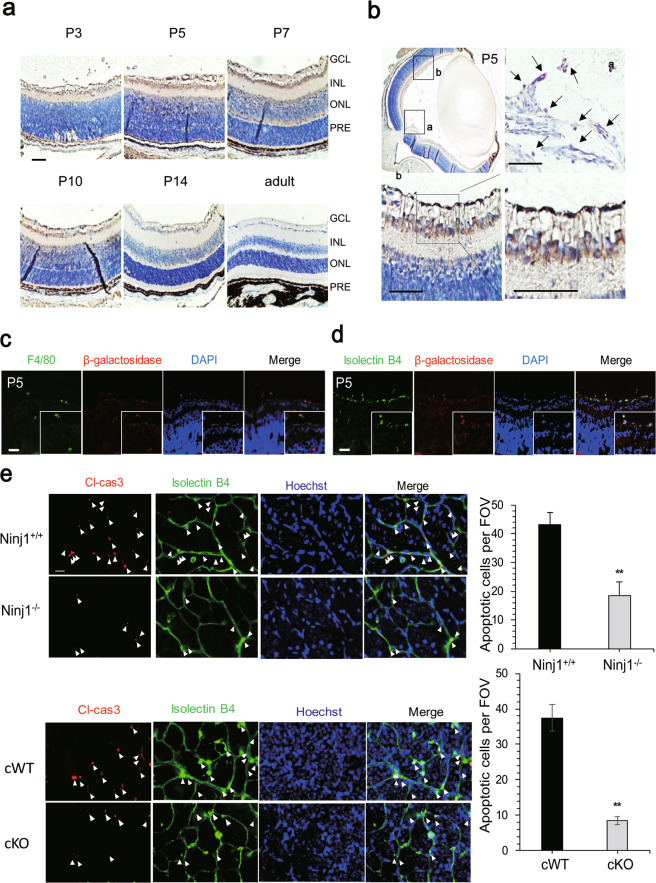
Ninj1-expressing macrophage mediates retinal endothelial cell apoptosis. **a** Expression patterns of Ninj1 using β-galactosidase, a reporter gene in retina of Ninj1^tm1a/+^ mice (P3, 5, 7, 14, and adult). Scare bar, 0.05 mm. **b** P5 whole-eye tissues of Ninj1^tm1a/+^ mice; **a** is the high-magnification image of a hyaloid vessel, **b** is the retinal blood vessel. Scare bar, 0.05 mm. **c** Double staining with antibodies for F4/80 (green) and β-galactosidase (red) in retina of Ninj1^tm1a/+^ mice (P5). White arrows indicate macrophages. Scale bar, 20 μm. **d** Double staining with antibodies for isolectin B4 (green) and β-galactosidase (red) in retina of Ninj1^tm1a/+^ mice (P5). **e** Cleaved caspase-3 (Cl-cas3, red) and isolectin B4 (green, endothelial cell) staining was used to identify the number of apoptotic endothelial cells in wild-type and Ninj1^−/−^ mice. Intensely stained, cleaved caspase-3-expressing cells (white arrowheads) within vessels were apparent. Nuclei (blue) were stained using Hoechst 33342, trihydrochloride trihydrate (Life Technologies). Scale bars, 50 μm. Graph indicates the intensity of cleaved caspase-3 in eye of Ninj1^+/+^ and Ninj1^−/−^ mice. Each bar represents mean ± S.D. (*n* = 8); ^**^P < 0.01, Ninj1^+/+^ vs. Ninj1^−/−^ mice. **f** Cleaved caspase-3 (Cl-cas3, red) and isolectin B4 (green, endothelial cell) staining was used to identify the number of apoptotic endothelial cells in conditional wild-type (cWT, Ninj1^fl/fl^; Lys-Cre^−/−^) and macrophage-specific conditional Ninj1 KO (cKO, Ninj1^fl/fl^; Lys-Cre^+/+^) mice. Intensely stained, cleaved caspase-3-expressing cells (white arrowheads) within vessels were apparent. Nuclei (blue) were stained using Hoechst 33342. Scale bars, 50 μm. Graph indicates the intensity of cleaved caspase-3 in eye of cWT and cKO mice. Each bar represents mean ± S.D. (*n* = 8); ^**^P < 0.01, Ninj1^+/+^ vs. Ninj1^−/−^ mice.

To elucidate the role of Ninj1 in retinal vascular development, we examined the spatiotemporal expression of Ninj1 in Ninj1^tm1a/+^ mice carrying a β-galactosidase trapping the cassette between exons 1 and 2 of Ninj1. β-Galactosidase was detectable in the retina from P3 to P14, but not in adult Ninj1^tm1a/+^ mice (Fig. 2a). β-Galactosidase-positive cells were present near the retinal vessels or ECs themselves (Fig. 2b). To identify the cell type of β-galactosidase-expressing cells, two markers, F4/80 (a macrophage marker) and isolectin B4 were used. The immunofluorescent signal of β-galactosidase strongly merged with F4/80 (Fig. 2c) but weakly merged with isolectin B4 (Fig. 2d), suggesting that Ninj1 was mainly expressed in macrophages and weakly in retinal VECs during postnatal development. This finding is different from previous reports stating that Ninj1 was only expressed in macrophages and only during the hyaloid regression [3]. Because macrophages could induce the apoptosis of ECs and exhibit phagocytic activity in both the vitreous compartment and the subretinal space [29], we investigated the changes in cleaved caspase-3 expression in Ninj1-deficient mice and found that the number of cleaved caspase-3-positive apoptotic ECs decreased compared to that in wild-type mice (Fig. 2e). To determine whether this cell death was caused by Ninj1-expressing macrophages, we examined the expression of cleaved caspase-3 in macrophage-specific conditional Ninj1 KO (cKO, Ninj1fl/fl; Lys-Cre^+/+^) mice, in which F4/80-positive macrophages did not express Ninj1 (Supplementary Fig. S1). The number of cleaved caspase-3-positive apoptotic ECs decreased significantly in Ninj1-deficient mice compared to that in conditional wild-type (cWT) mice (Ninj1^fl/fl^; Lys-Cre^−/−^) (Fig. 2f). Taken together, these data suggest that Ninj1 is expressed in macrophages and induces apoptosis of RECs during postnatal vascular development.

### MicroRNA-125a-5p is a novel negative regulator of Ninj1

**Fig. 3:**
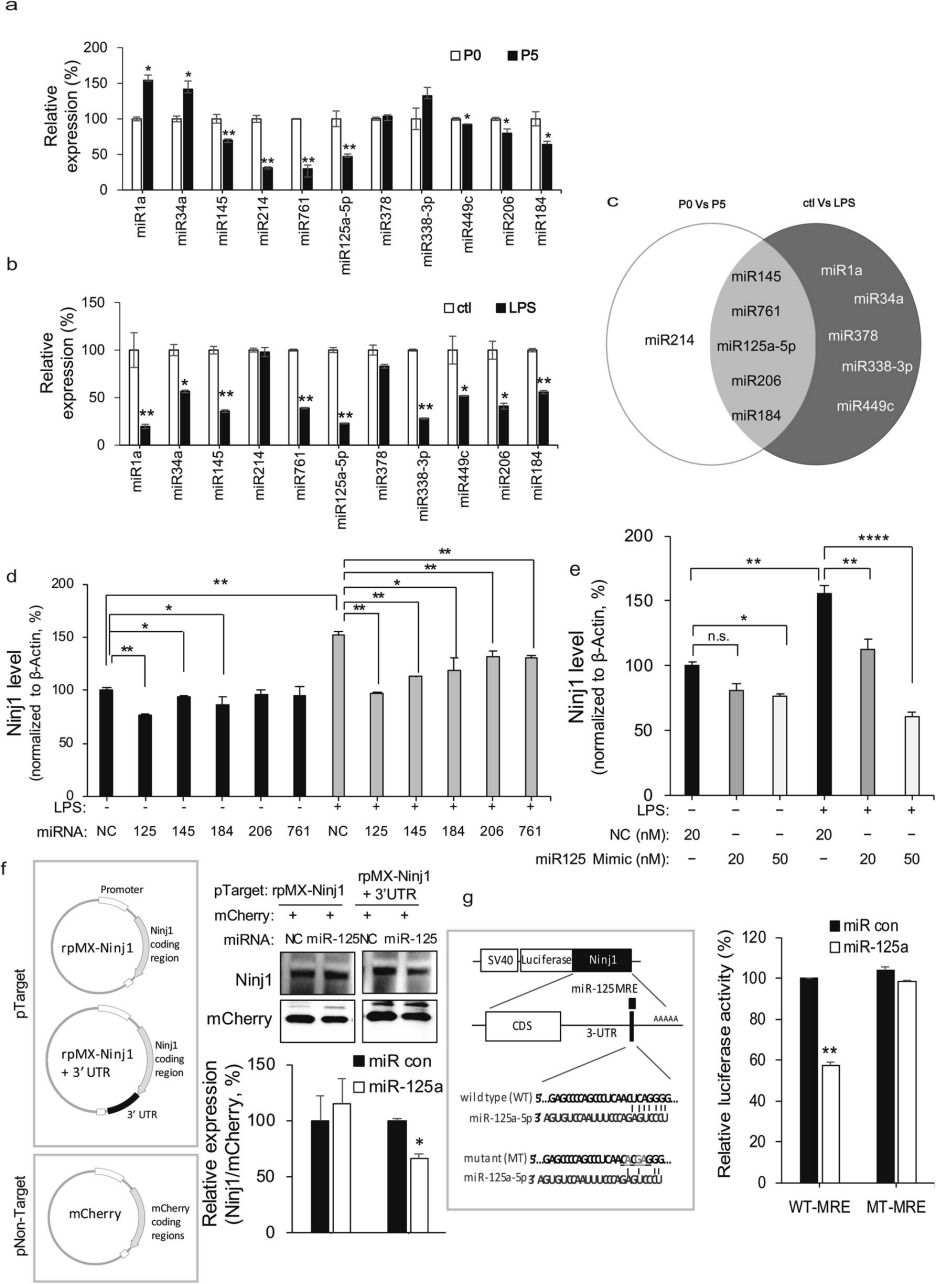
miR-125a-5p regulated Ninj1 expression. **a** The expression of miRNAs was determined by total RNA from eyes of P0 and P5 wild-type. Each bar represents mean ± S.D. (*n* = 5); ^*^P < 0.05, ^**^P < 0.01, eye of P0 mice vs. eye of P5 mice. **b** The expression of miRNAs was determined by total RNA in LPS-induced macrophages. Each bar represents mean ± S.D. (*n* = 5); ^*^P < 0.05, ^**^P < 0.01, vehicle group vs. LPS-induced group. **c** Venn diagrams based on real-time-PCR results showing the intersection between upregulated miRNAs in P5 and upregulated miRNAs in LPS-induced macrophages. **d** 20 nM miR-125a-5p, miR-145, miR-184, miR-206, and miR-761 mimics were transfected into Raw 264.7 cells. The negative and positive controls were transfected with 20 nM siRNA scramble. mRNA level of Ninj1 was analyzed by real-time PCR after LPS stimulation in RAW 264.7 cells. Each bar represents mean ± S.D. (*n* = 4); ^++^*P* < 0.01, vehicle group vs. LPS-induced group; ^**^P < 0.01, LPS-induced group vs. miR mimic-treated group. **e** RAW 264.7 cells transfected with two concentrations of miR-125a-5p mimic (20 and 50 nM). Negative and positive controls were transfected with 20 nM siRNA scramble. The mRNA level of Ninj1 was analyzed by real-time PCR after LPS stimulation in RAW 264.7 cells. Each bar represents mean ± S.D. (*n* = 4); ^**^P < 0.01, vehicle group vs. LPS-induced group;^+^*P* < 0.05, ^++^*P* < 0.01, LPS-induced group vs. miR-125a-5p-mimic-treated group. **f** The Ninj1 expression plasmids with or without 3′-UTR are shown (left). These plasmids and pmCherry-C1 empty vector were co-transfected with miR-125a-5p mimic into HEK 293 cells. The negative control (NC) was transfected with 20 nM siRNA scramble. After 2 days, the protein level of exogenously expressed Ninj1 and mCherry was determined by western blot analyses (right). Ninj1 expression level was normalized with mCherry level relative to that of control group (no transfection). Each bar represents mean ± S.D. (*n* = 4); ^*^P < 0.05; ^**^P < 0.01. **g** Outline of luciferase reporter assay for validating the interaction of miR-125a-5p with the 3′ UTR of Ninj1. The putative miR-125a-5p–binding site (wild-type or mutant) in the transcript of Ninj1 (left) was predicted by the miRanda algorithm. Wild-type or mutant Ninj1 3′-UTR were cloned into the pRL-TK Vector, as 3′ fusions to the luciferase gene. HEK 293 cells were transfected with the indicated miRNA mimics and luciferase vectors, sequentially. Luciferase activity was assayed 48 h later. Renilla luciferase activity was normalized with firefly luciferase level relative to that of control group. Each bar represents mean ± S.D. (*n* = 4); ^*^P < 0.05; ^**^P < 0.01.

Next, we attempted to identify miRNAs that bind to the UTR of Ninj1 and regulate its expression. First, we screened the potential miRNAs with binding sites in the 3′-untranslated region (3′-UTR) of Ninj1 using the miRanda algorithm [30]. Assuming that Ninj1 modulators will show a pattern opposite to that of Ninj1, we compared the expression levels of miRNAs under two conditions (P0 vs. P5 and LPS-activated inflammation), under which the expression of Ninj1 was notably increased. The expression levels of microRNA-145 (miR-145), miR-214, miR-761, miR-125a-5p, miR-449c, miR-206, and miR-184 were significantly reduced at P5 compared to those at P0 (Fig. 3a). When treated with LPS, the expression levels of miR-1a, miR-34a, miR-145, miR-761, miR-125a-5p, miR-338-3p, miR-449c, miR-206, and miR-184 were significantly decreased compared to those in the control group (Fig. 3b). The miRNAs showing decreased expression in both conditions were miR-145, miR-761, miR-125a-5p, miR-206, and miR-184 (Fig. 3c). To assess the inhibitory role of the selected miRNAs, we synthesized miRNA mimics (miR-mimics) corresponding to each miRNA. When RAW 264.7 cells were transfected with miR-mimics, all reduced Ninj1 expression mediated by LPS (Fig. 3d). Because miR-125a-5p mimic showed the most potent inhibitory effect on Ninj1 expression in a dose-dependent manner (Fig. 3e), subsequent experiments were conducted using miR-125a-5p mimic only. To verify whether miR-125a-5p is a novel regulator of Ninj1 and acts on the 3′-UTR of Ninj1, we performed two additional assays. First, we constructed Ninj1 expression vectors with or without the 3′-UTR (pTarget) and co-transfected them with pmCherry-C1 vector (pNon-Target) in HEK293 cells. As shown in Fig. 3f, miR-125a-5p mimic downregulated only the expression level of exogenous Ninj1 with 3′-UTR, but not mCherry protein and exogenous Ninj1 without 3′-UTR, suggesting that miR-125a-5p acts as a negative regulator of Ninj1 through the 3′-UTR. Next, we confirmed the binding of miR-125a-5p to miRNA-responsive element (MRE) within the site of the 3′-UTR using a luciferase reporter, in which a putative MRE site was downstream of the luciferase. When miR-125a-5p mimic was co-transfected with wild-type MRE-luciferase (WT-MRE), miR-125a-5p suppressed the luciferase activity by approximately 58% (*P* < 0.01) (Fig. 3g). However, the inhibition of luciferase activity mediated by miR-125a-5p mimic was abolished when a 3-base mismatch mutation was introduced into the MRE within the 3′-UTR (MT-MRE), indicating that the predicted MRE is critical for the specific binding of miR-125a-5p to the 3′-UTR of Ninj1 mRNA. These data suggest that miR-125a-5p is a novel regulator of Ninj1.

### MicroRNA-125a-5p inhibited Ninj1-mediated activation of macrophages in inflammatory conditions

**Fig. 4:**
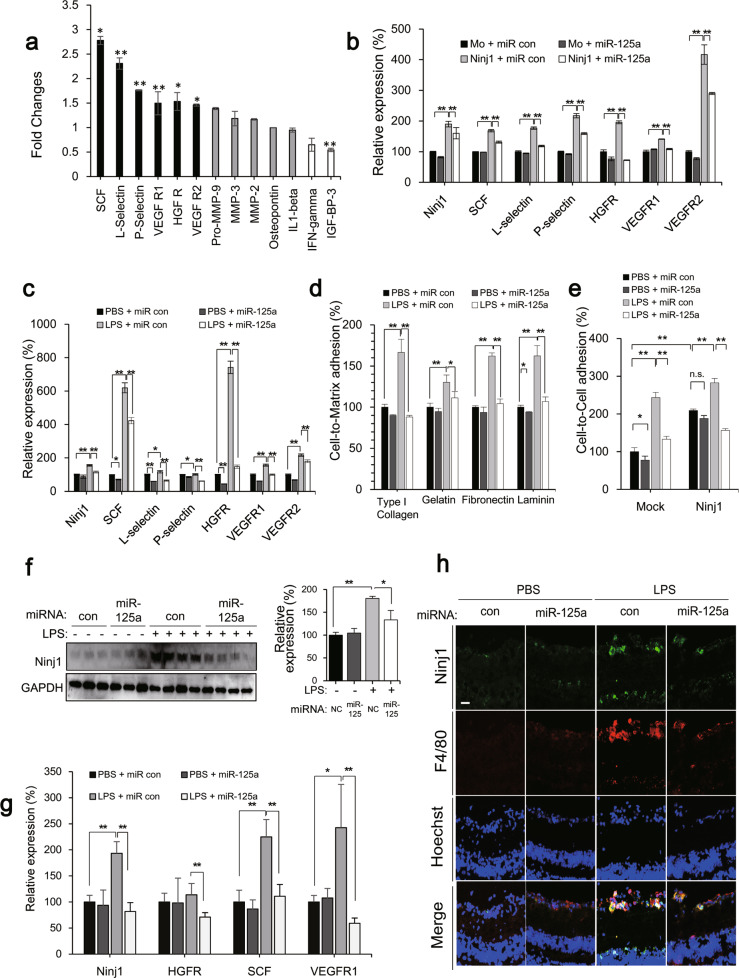
miR-125a-5p regulated retinal inflammation through Ninj1-mediated macrophage activity. **a** Quantification of chemokines and cytokines levels in pMXs-IRES-GFP-Ninj1 (rpMX-Ninj1) stably transfected RAW 264.7 cell lines. The bar graph summarizes significant differences in expression of cytokine-related genes using conditioned media of rpMX mock (control, rpMX-MO) and rpMX-Ninj1 stable RAW 264.7 cell lines. Each bar represents mean ± S.D. (*n* = 4); ^*^P < 0.05; ^**^P < 0.01. **b** Quantification of Ninj1 and cytokines mRNA expression levels in miR-125a-5p mimic (20 nM)-transfected Ninj1 stable RAW 264.7 cell line. Negative and positive controls were transfected with 20 nM siRNA scramble. mRNA levels of Ninj1 and its target genes were analyzed by real-time PCR in stable cell lines. Each bar represents mean ± S.D. (*n* = 4); ^**^P < 0.01, rpMX-MO group vs. rpMX-Ninj1 group; ^++^*P* < 0.01, rpMX-Ninj1 group vs. miR-125a-5p-mimic-treated rpMX-Ninj1 group. **c** Quantification of Ninj1 and cytokines mRNA expression levels in 20 nM miR-125a-5p-mimic-transfected RAW 264.7 cells. Negative and positive controls were transfected with 20 nM siRNA scramble. mRNA levels of Ninj1 and its target genes were analyzed by real-time PCR after LPS stimulation in RAW 264.7 cells. Each bar represents mean ± S.D. (*n* = 4); ^**^P < 0.01, vehicle group vs. LPS-induced group; ^++^*P* < 0.01, LPS-induced group vs. miR-125a-5p-mimic-treated group. **d** Quantification of cell-to-extracellular matrix (ECMs) adhesion using RAW 264.7 cells, in which each well was coated with 5 μg/ml fibronectin, 10 μg/ml type I collagen, 10 μg/ml laminin, or 5 μg/ml gelatin. Adhesion values were expressed relative to the adhesion of the vehicle-LPS group and LPS plus miR-125a-5p-transfected group, normalized to 100%. Each bar represents mean ± S.D. (*n* = 4); ^*^P < 0.05; ^**^P < 0.01. **e** Quantification of cell-to cell adhesion between mouse brain capillary endothelial cell 4 (MBEC4) and RAW 264.7 cells with either LPS (1 μg/ml) alone or miR-125a-5p-mimic-transfected group. Each bar represents mean ± S.D. (*n* = 4); ^*^P < 0.05; ^**^P < 0.01. **f** Protein expression of Ninj1 in eye lysates after injection of 330 μg/kg of LPS and LPS plus miR-125a-5p in C57BL/6 mice (7 weeks). Each bar represents mean ± S.D. (*n* = 3 or 4); ^*^P < 0.05; ^**^P < 0.01. **g** Quantification of Ninj1 and cytokines mRNA expression of Ninj1 and its target genes in eye lysates after injection of 330 μg/kg of LPS and LPS plus miR-125a-5p in C57BL/6 mice (7 weeks). Values were normalized to 100% at the expression level of control (PBS + miR con injected) mice. Each bar represents mean ± S.D. (*n* = 8); ^*^P < 0.05; ^**^P < 0.01. **h** Double staining with antibodies for Ninj1 (green) and F4/80 (red) in retina. Ninj1^+^F4/80^+^ cells were appeared only in LPS-inflamed retina and were disappeared in miR-125a-5p-mimic-treated group.

Ninj1 is known to play an important role in CNS inflammation [31–33], and recent bioinformatics analyses have shown that the expression of Ninj1 is closely linked to the expression of pro-inflammatory proteins and proangiogenic factors [34]. These have prompted us to investigate whether Ninj1 affects inflammatory mediators such as stem cell factor (SCF) and VEGF receptor-1 (VEGFR1) using a cytokine array. Ninj1-overexpressing RAW 264.7 macrophages showed notably higher levels of SCF, L-selectin, P-selectin, hepatocyte growth factor receptor (HGFR), VEGFR1, and VEGFR2 (Fig. 4a). The miR-125a-5p mimic significantly reduced the SCF, L-selectin, P-selectin, HGFR, VEGFR1, and VEGFR2 expression levels, which were increased by Ninj1 (Fig. 4b). Next, we determined whether miR-125a-5p mimic regulated the inflammatory conditions induced by LPS. As expected, miR-125a-5p mimic exerted a similar effect on the increase in SCF, L-selectin, P-selectin, HGFR, VEGFR1, and VEGFR2 levels mediated by LPS (Fig. 4c). Because Ninj1 mediates cell-to-ECM and cell-to-cell adhesion, we examined the effects of miR-125a-5p mimic on the adhesion of RAW 264.7 cells. The miR-125a-5p mimic effectively suppressed the enhanced adhesion of RAW 264.7 cells to ECMs, such as collagen type I, fibronectin, laminin, and gelatin, after LPS stimulation (Fig. 4d). The inhibitory effect of miR-125a-5p was confirmed in bone marrow-derived macrophages (BMDM) (Supplementary Fig. S2). Additionally, miR-125a-5p mimic significantly reduced the adhesion of macrophages to ECs, which was increased by LPS (Fig. 4e). We further investigated whether miR-125a-5p mimic could regulate Ninj1 expression levels and Ninj1-mediated macrophage activation using an endotoxin-induced inflammation model. Upon LPS stimulation, the expression levels of the Ninj1 were 3.8-fold higher than those in the control group, and miR-125a-5p mimic reduced the increase in Ninj1 levels by 2.9-fold (Fig. 4f). Furthermore, upregulation of pro-inflammatory factors, such as SCF and VEGFR1, by LPS was significantly decreased in miR-125a-5p-mimic-treated group (Fig. 4g). Next, we examined the effect of miR-125a-5p mimic on the recruitment of Ninj1-expressing macrophages into the retina through double staining of Ninj1 (green) and F4/80 marker (red). In LPS-inflamed retinas, the number of Ninj1-expressing macrophages increased; miR-125a-5p mimic abolished the increase in recruitment of macrophages (Fig. 4h). These data suggested that miR-125a-5p mimic decreased the levels of pro-inflammatory factors and infiltration of macrophages through Ninj1 downregulation during LPS-induced retinal inflammation.

### MicroRNA-125a-5p attenuated diabetic retinal impairments through Ninj1 downregulation

**Fig. 5:**
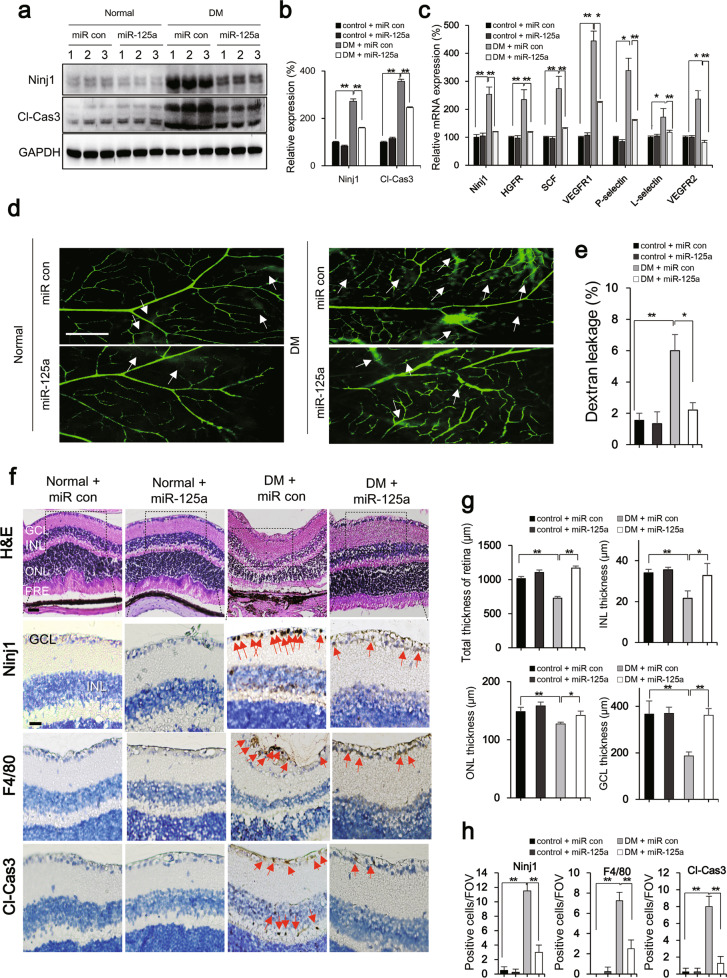
miRNA 125a-5p mediated diabetic retinal impairment through Ninj1-mediated activation of macrophages. miR-125a-5p mimic (miR-125a) and scramble siRNA (miR con) were intravitreally administered into a diabetic C57BL/6 mouse model. **a** The effect of miRNA 125a-5p on Ninj1 and cleaved caspase-3 (Cl-Cas3) protein expression in the retina of diabetic mice was analyzed by western blotting. **b** Quantification of Ninj1 and Cl-cas3 in the retina of normal and diabetic mice. Each bar represents mean ± S.D. (*n* = 3); ^*^P < 0.05; ^**^P < 0.01. **c** The effect of miRNA 125a-5p on mRNA expression levels of Ninj1 and inflammatory cytokines in the retina of diabetic mice. Each bar represents mean ± S.D. (*n* = 4); ^*^P < 0.05; ^**^P < 0.01. **d** The effect of miRNA 125a-5p on blood–retinal barrier leakage using FITC-dextran. FITC-dextran was injected into tail vein of mice. The retina was isolated and then viewed under a fluorescence microscope. Representative images of flat-mounted retina show extravasated FITC-dextran. **e** Quantification of dextran leakage. Each bar represents mean ± S.D. (*n* = 8 mice per condition); ^*^P < 0.05, ^**^P < 0.01. **f** The effect of miRNA 125a-5p on the infiltration of Ninj1-positive leukocytes. Representative H&E staining and immunostaining images using anti-Ninj1, anti-F4/80, and anti-Cl-Cas3 antibodies. Scale bars, 0.005 mm. **g** The effect of miRNA 125a-5p on retinal integrity. Histogram showing total thickness of retina, inner nuclear layer (INL), outer nuclear layer (ONL), and ganglionic cell layer (GCL) in wild-type and STZ-induced diabetic mice. **h** Bar graph indicates the number of Ninj1, F4/80, and Cl-Cas3-positive cells per field of view (FOV). All values are mean ± S.D. for each group (*n* = 8). ^*^P < 0.05.

Finally, we investigated the effect of miR-125a-5p mimic on DR. In DR, macrophages migrate to the site of inflammation and mediate retinal vessel pruning and phagocytosis, resulting in loss of integrity of retinal vasculature [35]. Thus, we investigated whether miR-125a-5p mimic prevented retinal impairment in an STZ-induced DR in C57BL/6 mice. Several diabetic indicators, such as body weight, fasting blood glucose, and hemoglobin A1c (HbA1c) were measured, and diabetes was confirmed (Table 1). In diabetic retinas, Ninj1 and Cl-cas3 levels were increased 2.27-fold and  3.56 fold compared to that in the normal group, respectively (Fig. 5a, b), and the expression level of pro-inflammatory factors was notably increased (Fig. 5c). However, miR-125a-5p mimic significantly decreased the increase in Ninj1 levels and pro-inflammatory factors mediated by Ninj1. Next, we examined whether miR-125a-5p mimic could inhibit blood vessel leakage in diabetes using fluorescein angiography. Extravasation of fluorescein isothiocyanate-conjugated dextran (FITC-dextran) appeared in the DR; this leakage was suppressed in the retinas of contralateral eyes injected with miR-125a-5p mimic (Fig. 5d, e). Additionally, several Ninj1-positive and F4/80-positive macrophages were observed in DR, which was abrogated in retinas treated with miR-125a-5p mimic (Fig. 5f). In addition, we verified the effectiveness of miRNA-125a-5p in ICR mice to confirm that the protective effect of miRNA-125a-5p is not specific to C57BL/6 mice. As expected, miRNA-125a-5p inhibited the expression of Ninj1 and Cl-cas3, reduced expression of inflammatory cytokines and significantly reduced the leakage of FITC-dextran (Supplementary Fig. S3) in STZ-induced diabetes in ICR mice. Finally, we investigated whether miR-125a-5p mimic recovered the integrity of the retinal layer by measuring the layer thickness in STZ-induced DR. Hematoxylin and eosin (H&E) staining revealed that total thickness of retina and thickness of ganglionic cell layer, inner nuclear layer, and outer nuclear layer were lower in the diabetic group than in the control group (Fig. 5g). However, miR-125a-5p mimic notably reversed retinal atrophy in the DR (Fig. 5g). Collectively, these data suggest that miR-125a-5p attenuates diabetic retinal impairment through the suppression of Ninj1 and pro-inflammatory factors.

**Table 1:** Comparison of body weight, blood glucose, and HbA1c in nondiabetic and diabetic C57BL/6 mice.

	Nondiabetic		Diabetic		*P* value
	miR con	miR-125a	miR con	miR-125a	
Body weight (g)	38.49 ± 1.03	39.52 ± 0.68	19.58 ± 2.80***	16.20 ± 2.13***	<0.001
Blood glucose (mg/dl)	144 ± 21.44	134.14 ± 7.21	451.44 ± 90.67***	455.8 ± 56.80***	<0.001
HbA1c (%)	3.73 ± 0.11	3.98 ± 0.04	9.07 ± 0.11***	8.97 ± 0.08***	<0.001

miR-125a-5p mimic (miR-125a) and scramble siRNA (miR con) were intravitreally administered into a diabetic C57BL/6 mouse model. Note: All values are mean ± S.D. for each group (*n* = 8); ^***^P < 0.001 compared with that of the nondiabetic scramble siRNA (miR con)-treated group.

## Discussion

The current world prevalence of diabetes mellitus is over 300 million [36]. Overall, 35% of diabetes patients with poor control experience DR within 20 years, which accounts for 5% of blind individuals worldwide [37]. Angiogenesis is the main cause of irreversible blindness, and many angiogenesis antagonists have been developed [38]. Bevacizumab has been developed as a tumor angiogenesis inhibitor [39], and is also used in DR and AMD off-label [40]. However, bevacizumab has shown various adverse effects, such as perforation [39]. Therefore, it is necessary to develop novel therapeutic agents for angiogenesis.

Inflammation is another key factor in DR [19], and many abnormalities underlying DR are related to inflammation. Levels of pro-inflammatory mediators, such as VEGF and IL-6, are abnormally increased in the retinas of DR patients [41]. Additionally, anti-inflammatory agents, such as non-steroidal anti-inflammatory drugs, show beneficial effects in DR [19]. Furthermore, inflammation and angiogenesis are closely associated with and promote each other [42]. Inflammation increases the permeability of retinal vessels and creates a pathological microenvironment. Therefore, the crosstalk between angiogenesis and inflammation could be a promising target for DR.

Ninj1 mediates cell–cell communication in pulmonary fibrosis, MS, and atherosclerosis [31–33]. A cohort study of idiopathic pulmonary fibrosis patients revealed that Ninj1 expression was increased in fibrosis patients compared to that in normal patients; this pattern was also observed in a mouse IPF model, in which Ninj1 mediated alveolar epithelial cell and macrophage interaction [31]. In CNS inflammation, leukocytes upregulate Ninj1 to increase cell adhesion and transmigration [5, 6]. Recently, it has been shown that a number of macrophages exist in the vitreous of diabetes patients [43], and DR is characterized by the existence of immature vascular walls and increased leukocyte infiltration [44]. Additionally, Ninj1 was significantly upregulated in both diabetes patients and diabetic mouse models [8, 45], and bioinformatics analysis revealed that the expression level of Ninj1 was significantly correlated with that of both proangiogenic genes and cell adhesion molecules in THP1 macrophages [34], suggesting the pathophysiological implications of Ninj1 in diabetes. Herein, we observed a significant increase in Ninj1 expression together with the expression of proangiogenic factors, such as SCF and VEGFR1, in the diabetic retina (Fig. 5c); Ninj1 mediated the infiltration of macrophages into the retina (Fig. 5f). These results are consistent with reports that Ninj1-neutralizing antibodies can protect ECs from diabetes [45]. To effectively modulate Ninj1 expression under pathological conditions, we identified miR-125a-5p as the key modulating factor (Fig. 3). It is known that the expression level of miR-125a-5p is reduced in diabetes [46] and that miR-125b-5p upregulation enhances insulin sensitivity by pancreatic β-cell activation [47]. Furthermore, miR-125a-5p silencing activates ECs and increases angiogenesis in vitro, and miR-125a-deficient zebrafish have shown increased angiogenesis [48]. These findings are consistent with our finding in that miR-125a-5p mimic blocked the entry of inflammatory cells into the retina (Fig. 5f) and attenuated vascular leakage (Fig. 5d, e). The inhibitory effects of miR125a-5p appear to be partly caused by the inhibition of SCF and VEGFR1 through Ninj1 downregulation. SCF destroys the adheren junctions of ECs and increases vascular permeability in DR [49]. Activation of VEGFR1 dysregulates VEGF-induced proliferation, migration, and barrier functions of ECs [50]. Furthermore, we found that miR-125-5p effectively inhibited the infiltration process of macrophages, thereby inhibiting inflammatory processes mediated by Ninj1 (Fig. 4). Taken together, Ninj1 could act as a promising therapeutic target for DR by regulating both angiogenesis and inflammation. However, the role of Ninj1 in angiogenesis may vary depending on the type of cell or tissue and the vascular status of the disease. Matsuki et al. reported that Ninj1 is highly expressed in capillary pericytes (cPCs), is weakly expressed in ECs of the thoracic aorta, and could act as an anti-angiogenic regulator of cPCs in ischemic models [51]. Additionally, Yin et al. showed that Ninj1 blockade enhanced penile angiogenesis in erectile dysfuntion [8] and Kim et al. reported that dodecamer peptide Ninj1 promotes angiogenesis during strokes [9]. Compared to dysregulated angiogenesis in DR, these lesions differ in that vasoreparative processes are caused by ischemic injury and the main players are pericytes instead of macrophages [52]. Pericytes are known to gradually disappear as diabetes develops [27], and we could not detect pericytes expressing Ninj1 in DR. On the other hand, macrophages are thought to play a major role in retinal vascular abnormalities, such as DR and ROP [53, 54]. Thus, the abnormal vascular inflammation or angiogenesis in which macrophages play a primary role could be managed by modulating Ninj1 expression.

It remains unclear whether the Ninj1 glycosylation pattern changes in DR and how ENT is processed during vascular inflammation and angiogenesis; however, our findings suggest that Ninj1 is a promising target for controlling inflammation and angiogenesis. Further investigation into the biological functions of Ninj1 and the molecular mechanisms involved in the development and breakdown of the retinal blood vessel network might provide novel insights into the field of DR and AMD treatment.

## Materials and methods

### Cell culture

Mouse brain capillary endothelial cell 4 (MBEC4), which were isolated from BALB/c mouse brain cortex and immortalized by SV40 transformation, and RAW 264.7 cells (from the Korea Cell Line Bank, Seoul, Korea) were cultured in Dulbecco’s modified Eagle’s medium (DMEM, Gibco BRL, MD, USA) supplemented with 10% fetal bovine serum (FBS, Gibco BRL) and 1% antibiotics (GenDEPOT, TX, USA) [5]. For the BMDMs, the BM was isolated from femurs and tibias and then cultured in RPMI 1640 medium (Gibco BRL) supplemented with 10% FBS for 3 days and differentiated in RPMI 1640 supplemented with macrophage-colony stimulating factor (20 ng/ml, R&D Systems, MN, USA) for 3 days. For adhesion assays, BMDM or RAW 264.7 cells were labeled with 5 μM carboxyfluorescein succinimidyl ester (CFSE), added to a MBEC4 monolayer or ECM, which was 5 μg/ml fibronectin (Invitrogen, CA, USA), 10 μg/ml type I collagen (BD Biosciences, CA, USA), 10 μg/ml laminin (Santa Cruz, TX, USA), and 5 μg/ml gelatin (Sigma-Aldrich, MO, USA) in 96-well plates and incubated for 20 min. After washing with PBS, the percentage of CFSE-labeled cells was quantified. Cells were transfected using Lipofectamine or the Neon Transfection System (Thermo Fisher, MA, USA) according to the manufacturer’s instructions. For the preparation of stable Ninj1 transfectants, 2 μg/ml puromycin (Sigma-Aldrich) was used.

### Expression vector cloning and transfection

Ninj1 cDNA, including coding sequences (CDS) with or without the 3′-UTR, was amplified by PCR with the forward primer 5′-GGGAATTCCGGCCGCACCATGGAGTCG-3′, reverse primer 5′-GTGGCGCCCCGGCAGTAG-3′ (CDS), or reverse primer 5′-ATCTCGAGAGCTTTA-TTGTCGTTCCTGATT-3′ (CDS + 3′-UTR). The PCR products were inserted into the pMXs-IRES-GFP expression vector (Cell BioLabs, CA, USA), between the EcoRI and XhoI sites. These plasmids and pmCherry-C1 empty vector (Clontech, Palo Alto, CA, USA) were co-transfected with either 20 nM siRNA scramble or 20 nM miR-125a-5p mimic into HEK 293 cells. The relative expression level of Ninj1 was analyzed by western blotting as previously described [55]. For the luciferase reporter assay, the 3′-UTR of Ninj1 possessing a putative miRNA-responsive element (MRE) was amplified by PCR using the following primers and cloned into the XbaI site downstream of the luciferase gene in the pRL-TK Vector (Promega) as previously described [56]. Ninj1 forward: 5′-GCTCTAGAACGCCCAGAGACTTTAAGGG-3′, wild-type Ninj1 reverse: 5′-AATCTAGAGGCCTGAAC-CCCTGAGTTGA-3′ with a putative binding site of miR-125a-5p, mutant Ninj1 reverse: 5′-AATCTAGAGGC-CTGAACCCTCGTGTTGA-3′ with a mutated binding site of miR-125a-5p. The nucleotide sequences of the constructed plasmids were confirmed by DNA sequencing (Macrogen, Daejeon, Korea).

### Generation of Ninj1-expressing stable cell lines and chemokine/cytokine antibody arrays

Raw 264.7 cells were transfected with pMXs-IRES-GFP-Ninj1 plasmid using Lipofectamine-3000 transfection reagent (Invitrogen) and cultured for an additional 2 days. The cells were selected for puromycin resistance (4 μg/ml) for 10 days, and maintained in medium containing 0.5 μg/ml pyromycin. The expression level of Ninj1 was analyzed by western blotting and immunofluorescence staining (Supplementary Fig. S4). At 80% confluence, the cells were cultivated in serum-free media for 2 days. The conditioned media was collected and applied to a RayBio mouse cytokine antibody array C1000 (RayBiotech, GA, USA) according to the manufacturer’s instructions. Data analysis was performed using RayBiotech Analysis Tools software.

### Animal maintenance

Ninjurin1-deficient C57BL/6J mice were maintained in the animal housing facility of Seoul National University according to the Committee for Care and Use of Laboratory Animals at Seoul National University (SNU-101011-1). The heterozygous Ninj1 tm1a mutant (Ninj1^tm1a/+^) mice were purchased from the Knockout Mouse Project (KOMP; University of California, Davis, USA) and ICR mice were purchased from Dae-Han Bio Inc. (Chungbuk, Korea). Ninj1^tm1a/+^ mutant mice were mated with Flpo mice (KOMP) to obtain heterozygous Ninj1 tm1c mice (Ninj1^tm1c/+^ or Ninj1^fl/+^ mice), which do not have a trapping cassette. To generate macrophage-specific Ninj1 KO mice, Ninj1^fl/fl^ mice were bred with heterozygous Lysozyme 2–Cre (Lyz2-cre^+/−^, Jackson Laboratory, Bar Harbor, ME, USA) as previously described [57]. The mice were housed under a 12 h light–dark cycle and had access to food and water ad libitum. All animal experiments were approved by the Committee on Animal Research at Inje University (Inje 2016-018) and Sungkyunkwan University (SKKUIACUC2021-11-19-2, SKKUIACUC2021-09-23-1).

### Induction of diabetes in mice

Six-week-old, male C57BL/6J mice were administered 180 mg/kg streptozotocin (STZ, Sigma) freshly dissolved in 50 mM sodium citrate buffer (pH 4.5) by a single intraperitoneal injection as previously reported [58]. The animals were randomly and blindly divided into four groups (eight mice per group). Control groups received vehicle (citrate buffer) only. On the 4th day after STZ injection, mice with blood glucose levels of 300 mg/dl or more were regarded as diabetic. After 3 weeks, 20 μM of miRNA mimic 125a-5p was prepared using Lipofectamine 3000 and injected intravitreally using a 34-gauge needle in STZ-induced diabetic C57BL/6 mice. Eight-week-old male ICR mice were intraperitoneally injected with 180 mg/kg STZ after 4 h of starvation. Next, 10% sucrose was administered instead of water for 3 days, and on day 4, mice with a fasting blood glucose level of 300 mg/dl were considered diabetic. After 10 weeks, 20 μM of miRNA mimic 125a-5p was prepared using Lipofectamine 3000 and injected intravitreally using a 34-gauge needle in STZ-induced diabetic ICR mice. Scrambled RNA (20 μM) was used as a negative control. Before STZ-induced diabetic mice were sacrificed, 100 μL of 70 kDa FITC-dextran (cat. no. FD70S) was injected intravenously. After 1 h of perfusion, the mice were sacrificed by inhalation of CO_2_, and their eyes were isolated and fixed in 4% PFA for 2 h at 20 °C. Flat-mounted retinas were observed using an Axiovert M200 microscope (Zeiss, Oberkochen, Germany) and the images were imported into ImageJ (1.47v, NIH, Bethesda, MD, USA). FITC-conjugated dextran was measured as the dextran area outside the vessels divided by the total measured area of the retina using ImageJ software, as previously described [59].

### Luciferase assay

HEK 293 cells were co-transfected with pRL-TK Renilla plasmid containing the 3′-UTR of Ninj1 and pGL3-control firefly luciferase vectors (as an internal control). After 24 h, miRNA mimics were transfected. At 2 days post-transfection, reporter assays were performed and relative luciferase activity was calculated by normalizing the ratio of Renilla/Firefly luciferase to that of negative control-transfected cells as previously described [56].

### miRNA target prediction and miRNA mimics preparation

The miRanda algorithm predicted 11 putative miRNA binding sites in the UTR region of mouse Ninj1, including miR-1a, miR-34a, miR-145a-5p, miR-214, miR-761, miR-125a-5p, miR-378, miR-338-3p, miR-449c, miR-206-3p, and miR-184-3p [30]. miRNA mimics (miR-125a-5p, #MSY0000135; miR-145a-5p, #MSY0000157; miR-184-3p, #MSY0000213; miR-761, #MSY0003893; and miR-206-3p, #MSY0000239) were purchased from Qiagen (Hilden, Germany) and transfected using Lipofectamine 3000 (Invitrogen, cat. no. L3000001), according to the manufacturer’s instructions.

### Immunohistochemistry and immunofluorescence staining

For histological evaluation, enucleated eyes were fixed in 4% paraformaldehyde overnight and then embedded in paraffin or OCT compound (Sakura-Finetek, MA, USA). Fixed tissues were cut into 4 μm-thick sections and mounted on microscope slides. The sections were used for H&E staining or immunostaining according to the standard protocol. Fluorescence-conjugated isolectin B4 (#I21411, Thermo Fisher) and antibodies against Ninj1 (custom-made antibodies prepared by Abfrontier, Seoul, Korea) [4], F4/80 (#MCA497G, AbD Sertotec, Oxford, UK), β-galactosidase (#AB1211, Millipore, MA, USA), and cleaved caspase-3 (#9661, Cell Signaling Technology, MA, USA) were used. Nuclei were counterstained with Hoechst 33342 (Life Technologies, Carlsbad, CA, USA). Images were obtained using an Axiovert M200 microscope (Zeiss, Iena, Germany). The random fields in each section were calculated using ImageJ software and the relative expression level of each protein was quantified according to integrated optical density from three independent experiments. *P* values were calculated using the log-rank test.

### Repetition and statistical analysis

All experiments were repeated more than three times. The results are expressed as mean ± S.D. All statistical tests were carried out using SPSS software (version 24; IBM, Armonk, NY, USA). Differences between two groups were evaluated using an unpaired Student’s *t* test and were considered to be statistically significant when *P* < 0.05. For immunohistochemical analyses, *P* values were calculated using the log-rank test.

### Other methods

Western blot analysis, RNA isolation, and real-time quantitative polymerase chain reaction (RT-qPCR) were performed according to the manufacturer’s protocol. See Supplementary methods for further details.

## Supplementary information

Change of authorship request form.

Supplemental information.

Reproducibility Checklist form.

Supplementary Figure S1.

Supplementary Figure S2.

Supplementary Figure S3.

Supplementary Figure S4.

## Data Availability

Data supporting the present study are available from the corresponding author upon reasonable request.
