# Supplementary material for: miR-125a-5p attenuates macrophage-mediated vascular dysfunction by targeting Ninjurin1
Source: Cell Death Differ. 2022 Jan 1;29(6):1199–210. doi: 10.1038/s41418-021-00911-y (PMC9177769; doi:10.1038/s41418-021-00911-y)
Supplement: Supplementary file 1 — Change of authorship request form. [file 41418_2021_911_MOESM1_ESM.pdf]

SPRINGER NATURE

Change of authorship request form - Journals (pre-acceptance)

Important information. Please read.

- This form should be used by authors to request any change in authorship (adding/deleting authors) including changes in corresponding authors. This form should not be used for name changes. Please fully complete all sections. Use black ink and block capitals and provide each author's full name with the given name first followed by the family name.

- By signing this declaration, all authors guarantee that the order of the authors are in accordance with their scientific contribution, if applicable as different conventions apply per discipline, and that only authors have been added who made a meaningful contribution to the work.

- Please note, in author collaborations where there is formal agreement for representing the collaboration, it is sufficient for the representative or legal guarantor (usually the corresponding author) to complete and sign the Authorship Change Form on behalf of all authors, **next to the added/removed author(s)**. **(Complete Section 3, followed by Section 6.)**
  In author collaborations where there is no formal agreement for representing the collaboration and **there are more than 10 authors**, one may sign for all, provided the signer appends correspondence that attests that each of the authors have agreed to the change **and the added/removed authors sign the form**. **(Complete Section 3, followed by Section 6.)**

- Please note, we cannot investigate or mediate any authorship disputes. If you are unable to obtain agreement from all authors (including those who you wish to be removed) you must refer the matter to your institution(s) for investigation. Please inform us if you need to do this.

- If you are not able to return a fully completed form within **30 days** of the date that it was sent to the author requesting the change, we may have to withdraw your manuscript. We cannot publish manuscripts where authorship has not been agreed by all authors (including those who have been removed).

- Incomplete forms will be rejected.

- Please return/upload this form, fully completed, to the Journals Editorial Office. The Journal and/or Publisher will consider the information you have provided to decide whether to approve the proposed change in authorship. We may decide to contact your institution for more information or undertake a further investigation, if appropriate, before making a final decision.

SPRINGER NATURE

Change of authorship request form - Journals (pre-acceptance)

Section 1: Please provide the current title of manuscript

Manuscript ID no.: CDD-21-0868RR

Title: miR-125a-5p attenuates macrophage-mediated vascular dysfunction by targeting Ninjurin1

Section 2: Please provide the previous authorship, in the order **shown on the manuscript before the changes were introduced. Please indicate the corresponding author by adding (CA) behind the name.**

	First name(s)	Family name	ORCID or SCOPUS id, if available
1st author	Su Jung	Hwang	
2nd author	Bum Ju	Ahn	
3rd author	Minwook	Shin	
4th author	Goo Taeg	Oh	
5th author	Kyu-Won	Kim	
6th author	Hyo-Jong	Lee	0000-0003-4826-7954
7th author			
8th author			
9th author			
10th author			

Please use an additional sheet if there are more than 10 authors.

SPRINGER NATURE Springer Nature is one of the world's leading global research, educational and professional publishers, created in May 2015 through the combination of Nature Publishing Group, Palgrave Macmillan, Macmillan Education and Springer Science+Business Media.

SPRINGER NATURE

Change of authorship request form - Journals (pre-acceptance)

Section 3: Please provide a **justification** for change. Please use this section to **explain** your reasons **for changing the authorship of your manuscript, e.g. what necessitated the change in** authorship? Please refer to the (journal) policy pages for more information about authorship. Please explain **why omitted authors were not originally included and/or why authors were removed on the submitted manuscript.**

Ye-Seul Song and Youngbin Choi were newly added, because they performed experimental procedures and data analysis during revision. Specifically, Y.C was in charge of the miRNA validation experiment, and YSSng was in charge of conditional KO mouse analysis.

Section 4: Proposed **new** authorship. Please provide your new authorship **list** in the order you would like **it** to appear **on the manuscript. Please indicate the corresponding author by** adding **(CA)** behind the name. **If the Corresponding Author has changed, please indicate the reason under section 3.**

	First name(s)	Family name (this name will appear in full on the final publication and will be searchable in various abstract and indexing databases)	Affiliated institute	E-mail address
1st author	Su Jung	Hwang	School of Pharmacy, Sungkyunkwan University	sama3575@naver.com
2nd author	Bum Ju	Ahn	College of Pharmacy, Seoul National University	bomb78@snu.ac.kr
3rd author	Minwook	Shin	College of Pharmacy, Seoul National University	shmiwo@gmail.com
4th author	Ye-Seul	Song	School of Pharmacy, Sungkyunkwan University	summerai6533@naver.com
5th author	Youngbin	Choi	School of Pharmacy, Sungkyunkwan University	zlot0315@gmail.com
6th author	Goo Taeg	Oh	Department of Life Sciences, Ewha Womans University	gootaeg@ewha.ac.kr
7th author	Kyu-Won	Kim	College of Pharmacy, Seoul National University	qwonkim@snu.ac.kr
8th author	Hyo-Jong	Lee	School of Pharmacy, Sungkyunkwan University	pharm79@skku.edu
9th author				
10th author				

Please use an additional sheet if there are more than 10 authors.

Section 5: Author contribution, Acknowledgement and Disclosures. Please use this section to provide a new disclosure statement and, if appropriate, **acknowledge any contributors who** have been removed as authors and ensure you state what contribution any new authors made **(if** applicable **per the journal or book (series) policy).** **Please ensure these are updated in your manuscript - after approval of the change(s) - as our production department will not transfer the information in this form to your manuscript.**

New acknowledgements:

Not applicable

New **Disclosures (financial and non-financial interests, funding):**

Conflict of Interest
The authors declare no competing interests.

Funding
This research was supported by the Basic Science Research Program (2019R1C1C1003558, H-JL) of the Ministry of Science, ICT and Future Planning through the National Research Foundation of Korea (NRF), the Ministry of Education, Science and Technology (2019R1A6A3A 01091754, SH) and the Bio-Synergy Research Project (NRF-2021M3A9C4000960, H-JL) of the Ministry of Science, ICT and Future Planning through the NRF.

New Author **Contributions statement (if applicable per the journal policy):**

SH performed experimental procedures, paper writing, and data analysis. Y-SS and YC performed experimental procedures, and data analysis. BA, MS, GO and KK performed mice experiments. H-JL supervised the entire project, designed the experiment, analyzed the data, revised the paper, and approved the final version of the paper for publication

State 'Not applicable' if there are no new authors.

SPRINGER NATURE

Change of authorship request form - Journals (pre-acceptance)

Section 6: Declaration of agreement. ***All*** **authors, unchanged, new and removed** ***must*** **sign this declaration.**
(NB: Please print the form, (docu)-sign and return/upload a scanned copy. Please note that signatures that have been inserted as an image file are acceptable as long as it is handwritten. Typed names in the signature box are unacceptable.) *** Please delete as appropriate. Delete all of the bold if you were on the original authorship list and are remaining as an author.**

	First name	Family name		Signature	Date
1st author	Su Jung	Hwang	I agree to the proposed new authorship shown in section 4 **/and the addition/removal*of my name to the authorship list** /and the proposed change in corresponding author	황 수 정	Nov 19, 2021
2nd author	Bum Ju	Ahn	I agree to the proposed new authorship shown in section 4 **/and the addition/removal*of my name to the authorship list** /and the proposed change in corresponding author	안 범주	Nov 19, 2021
3rd author	Minwook	Shin	I agree to the proposed new authorship shown in section 4 **/and the addition/removal*of my name to the authorship list** /and the proposed change in corresponding author	Minwook Shin.	Nov 19, 2021
4th authors	Ye-Seul	Song	I agree to the proposed new authorship shown in section 4 **/and the addition/removal*of my name to the authorship list** /and the proposed change in corresponding author	송예슬	Nov 19, 2021
5th author	Youngbin	Choi	I agree to the proposed new authorship shown in section 4 **/and the addition/removal*of my name to the authorship list** /and the proposed change in corresponding author	최영빈	Nov 19, 2021
6th author	Goo Taeg	Oh	I agree to the proposed new authorship shown in section 4 **/and the addition/removal*of my name to the authorship list** /and the proposed change in corresponding author	오 구 택	Nov 19, 2021
7th author	Kyu-Won	Kim	I agree to the proposed new authorship shown in section 4 **/and the addition/removal*of my name to the authorship list** /and the proposed change in corresponding author	김 규 원	Nov 19, 2021

SPRINGER NATURE Change of authorship request form - Journals (pre-acceptance)

	First name	Family name		Signature	Date
8th author	Hyo-Jong	Lee	I agree to the proposed new authorship shown in section 4 **/and the addition/removal*of my name to the authorship list** /and the proposed change in corresponding author	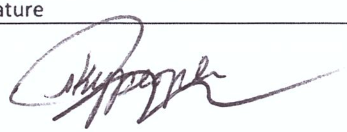	Nov 19, 2021
9th author			I agree to the proposed new authorship shown in section 4 **/and the addition/removal*of my name to the authorship list** /and the proposed change in corresponding author		
10th author			I agree to the proposed new authorship shown in section 4 **/and the addition/removal*of my name to the authorship list** /and the proposed change in corresponding author		

Please use an additional sheet if there are more than 10 authors.

In case of author collaborations with formal agreement:

	Name of consortium/consortia	First name	Family name		Signature	Date
Representative/legal guarantor				I agree to the proposed new authorship shown in section 4 **/and the addition/removal*of my name to the authorship list** /and the proposed change in corresponding author		

Both added/removed authors should complete the information in the first table under Section 6.

---- End of form ----

SPRINGER NATURE Springer Nature is one of the world's leading global research, educational and professional publishers, created in May 2015 through the combination of Nature Publishing Group, Palgrave Macmillan, Macmillan Education and Springer Science+Business Media.
