# Supplementary material for: miR-125a-5p attenuates macrophage-mediated vascular dysfunction by targeting Ninjurin1
Source: Cell Death Differ. 2022 Jan 1;29(6):1199–210. doi: 10.1038/s41418-021-00911-y (PMC9177769; doi:10.1038/s41418-021-00911-y)
Supplement: Supplementary file 2 — Supplemental information. [file 41418_2021_911_MOESM2_ESM.docx]

**Supplementary Figure Legends**

**Supplementary Fig. S1 The effect of macrophage-specific deletion of Ninj1 deletion on macrophage infiltration in developing retina at postnatal day eight (P8).** Double staining with antibodies for F4/80 (red) and Ninj1 (green) in conditional wild-type (cWT, Ninj1^fl/fl^; Lys-Cre^-/-^) and macrophage-specific conditional Ninj1 KO (cKO, Ninj1^fl/fl^; Lys-Cre^+/+^) mice. Nuclei (blue) were stained using Hoechst 33342. Scale bars, 100 μm.

**Supplementary Fig. S2 The effect of miR-125a-5p mimic on cell-to matrix adhesion of BMDMs.** Quantification of cell-to-matrix adhesion in 20 nM miR-125a-5p mimic-transfected bone marrow-derived macrophages (BMDMs). BMDMs suspended in serum-free media were added to each coated well with 5 μg/ml fibronectin, 10 μg/ml type I collagen, 10 μg/ml laminin, and 5 μg/ml gelatin, which are components of ECMs at 4 ℃ for 24 h. Non-adherent cells were removed by washing and the remaining adherent cells were quantified as described in the Material and Methods. Cell-to-matrix adhesion assays using BMDM cells with either LPS (1 μg/ml) alone or miR-125a-5p mimic-transfected group. Adhesion values are expressed relative to the adhesion of vehicle-LPS group and LPS plus miR-125a-5p transfected group, normalized to 100%. Each bar represents the mean ± S.D. (n = 6). *, P < 0.05; **, P < 0.01.

**Supplementary Fig. S3 miRNA 125a-5p mediated diabetic retinal impairment in ICR mice.** miR-125a-5p mimic and scramble siRNA were intravitreally administered into a diabetic ICR mouse model. a The effect of miRNA 125a-5p on Ninj1 and cleaved caspase-3 (Cl-Cas3) protein expression in the retina of diabetic mice was analyzed by Western blotting. b Quantification of Ninj1 and Cl-cas3 in the retina of normal and diabetic mice. Each bar represents the mean ± S.D. (n = 3). *, P < 0.05; **, P < 0.01. c The effect of miRNA 125a-5p on mRNA expression levels of Ninj1 and inflammatory cytokines in the retina of diabetic mice. Each bar represents the mean ± S.D. (n = 4). *, P < 0.05; **, P < 0.01. d The effect of miRNA 125a-5p on blood–retinal barrier leakage using FITC-dextran. FITC-dextran was injected into tail vein of mice. The retina was isolated and then viewed under a fluorescence microscope. Representative images of flat-mounted retina show extravasated FITC-dextran. e Quantification of dextran leakage. Each bar represents the mean ± S.D. (n = 8 mice per condition); *P < 0.05, **P < 0.01.

**Supplementary Fig. S4 The RAW. 264.7 cells stably expressing Ninj1.** RAW 264.7 cells was transfected with either rpMX mock vector (rpMX MO) or pMXs-IRES-GFP Ninj1 plasmid (rpMX Ninj1) and then treated with puromycin for 10 days to select Ninj1-stably transfected RAW 264.7 cells. **a** Immunoblotting of GFP and Ninj1 in stably transfected RAW 264.7 cells. Tubulin was used as an internal control. **b** Immunofluorescence images of GFP (green) and Ninj1 (red) in stably transfected RAW 264.7 cells. Nuclei (blue) were stained using Hoechst 33342. Scale bar, 30 μm.

**Supplementary Methods**

**Western blot analysis**

Samples for western blotting were lysed in lysis buffer containing 40 mM Tris-HCl (pH 7.4), 10 mM EDTA, 120 mM NaCl, 0.1% NP-40, 1 mM PMSF, and 2 μg/ml leupeptin. The lysates were resolved by 10% sodium dodecyl sulfate-polyacrylamide gel electrophoresis and transferred to nitrocellulose membranes. The membranes were blocked in Tris-buffered saline (10 mM Tris-Cl pH 7.4) containing 0.5% Tween-20 and 5% nonfat dry milk, incubated with the primary antibody in blocking solution overnight at 4 °C, washed, and incubated with the secondary antibody for 1h at 20 °C. Antibodies used for western blotting were: mCherry (#43590, Cell Signaling Technology), Tubulin (#sc-5286, Santa Cruz), GFP (#sc-8334, Santa Cruz), GAPDH (#sc-25778, Santa Cruz), and Ninj1 (Custom-made). The respective protein bands were detected using FUSION-SL4 chemiluminescence (Vilber).

**RNA isolation and real-time quantitative polymerase chain reaction (RT-qPCR)**

Total RNA was isolated from the samples using the TRIzol reagent (Invitrogen). Real-time PCR was performed using one-step qRT-PCR kits (Qiagen) and following primers (Bioneer, Daejeon, Korea): SCF (forward, 5′-GAATCTCCGAAGAGGCCAGAA-3′; reverse, 5′-GCTGCAACAGGGGGTAACAT-3′); HGFR (forward, 5′-GGAACTGGCTACTGCTCTGG-3′; reverse, 5′-GGTGGGAGCCTTCATTGTGA-3′); P-selectin (forward, 5′-TGGATCAACCTGCACCTTCC-3′; reverse, 5′-TGGATTGTCAGTGTCCCTGC-3′); L-selectin (forward, 5′-GTCCAAGGAGGACTGTGTGTGG-3′; reverse, 5′-ACACATTCTCCACGGC-CATT-3′); VEGFR1 (forward, 5′-CCATCTGACCAACCTCCGC-3′; reverse, 5′-CGCTCAAAGAGATAC-TCGCC-3′); VEGFR2 (forward, 5′-TTCACAGTCGGGTTACAGGC-3′; reverse, 5′-TCTCACAATTCTTC-GGCCCC-3′); Ninj1 (forward, 5′-ACTGAGGAGTATGAGCTCA-3′; reverse, 5′-TCCATTACAGGCTTC-TGGA-3′); actin (forward, 5′-AGAGGGAAATCGTGCGTGAC-3′; reverse, 5′-GGCCGTCAGGCAGCT-CATAG-3′). For miRNAs, miRNAs from dissected tissues or cultured cells were purified using the Hybrid-RTM miRNA isolation kit (GeneAll) according to the manufacturer’s instructions. The eluted 200 ng miRNAs were synthesized using the miScript reverse transcription kit (Qiagen) following the manufacturer’s recommendations. Real-time PCR was performed using the miScript SYBR Green PCR kit (Qiagen) with the target miRNA primers. For miRNA PCR, 200 ng of cDNA was diluted to 20 ng cDNA. miRNAs were quantified using U6 small RNA (normalization control). The miRNA primers used in this study are listed below and were purchased from Bioneer: miR-1a, 5′-TGGAATGTAAAGAAGTATGTAT-3′; miR-206-3p, 5′-TGGAATGTAAGGAAGTGTGTGG-3′; miR-184-3p, 5′-TGGACGGAGAACTGATAAGGGT-3′; miR-34a, 5′-TGGCAGTGTCTTAGCTGGTTGT-3′; miR-145a-5p, 5′-GTCCAGTTTTCCCAG-GAATCCCT-3′; miR-214, 5′-ACAGCAGGCACAGACAGGCAGT-3′; miR-761, 5′-GCAGCAGGGTGAA-ACTGACACA-3′; miR-125a-5p, 5′-TCCCTGAGACCCTTTAACCTGTGA-3′; miR-378, 5′-ACTGGACT-TGGAGTCAGAAGG-3′; miR-449c, 5′-AGGCAGTGCATTGCTAGCTGG-3′; miR-338-3p, 5′-TCCAGC-ATCAGTGATTTTGTTG-3′. Thermal cycling conditions were 95 °C for 15 min, followed by 40 cycles of 94 ℃ for 15s, 55 ℃ for 30s, and 70 ℃ for 30s. Cycle thresholds were calculated using an automatic baseline and a threshold of 0.1.
