# Supplementary figures and images for: miR-125a-5p attenuates macrophage-mediated vascular dysfunction by targeting Ninjurin1
Source: Cell Death Differ. 2022 Jan 1;29(6):1199–210. doi: 10.1038/s41418-021-00911-y (PMC9177769; doi:10.1038/s41418-021-00911-y)

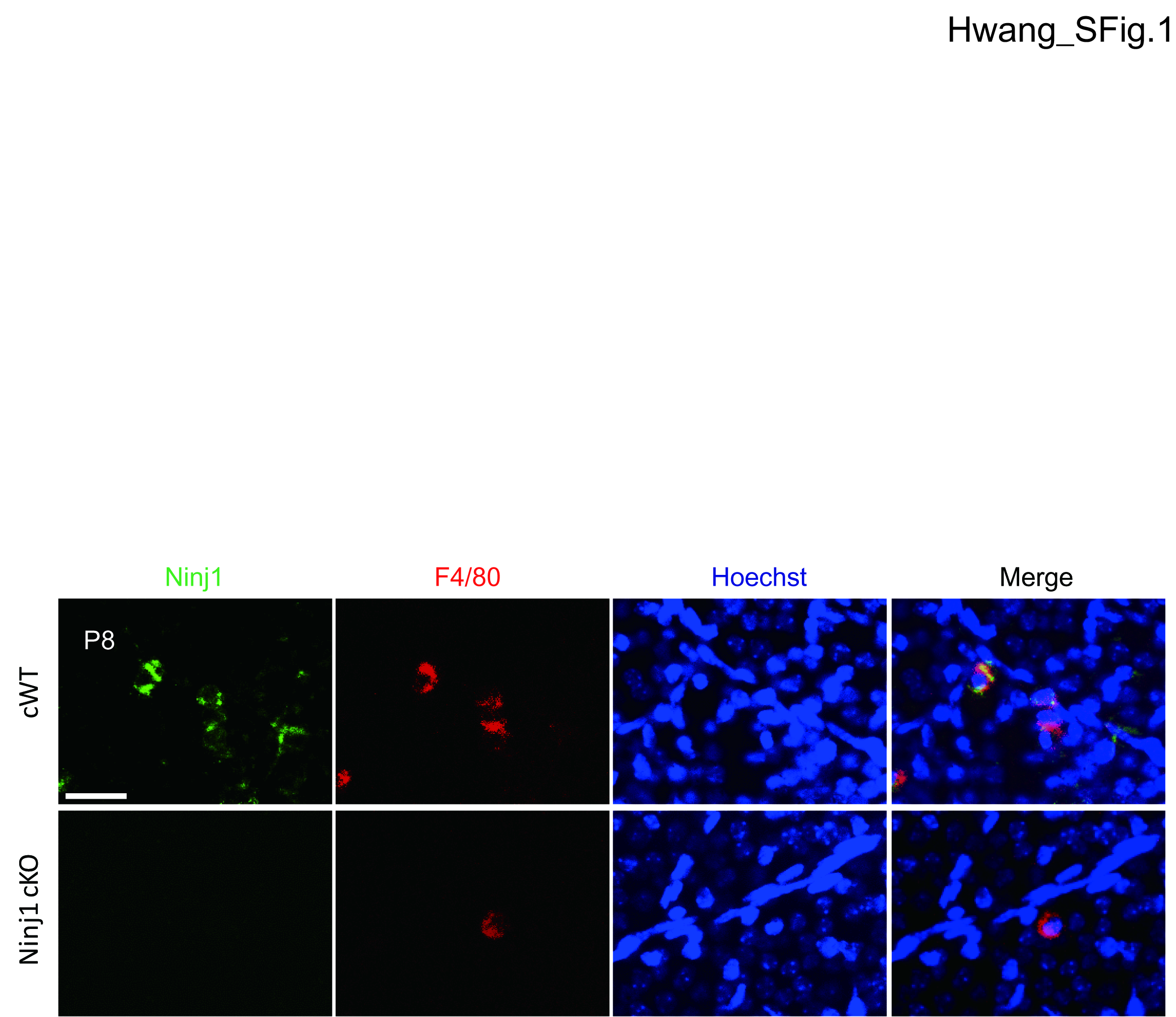

Supplement: Supplementary file 4 — Supplementary Figure S1. [file 41418_2021_911_MOESM4_ESM.tif]

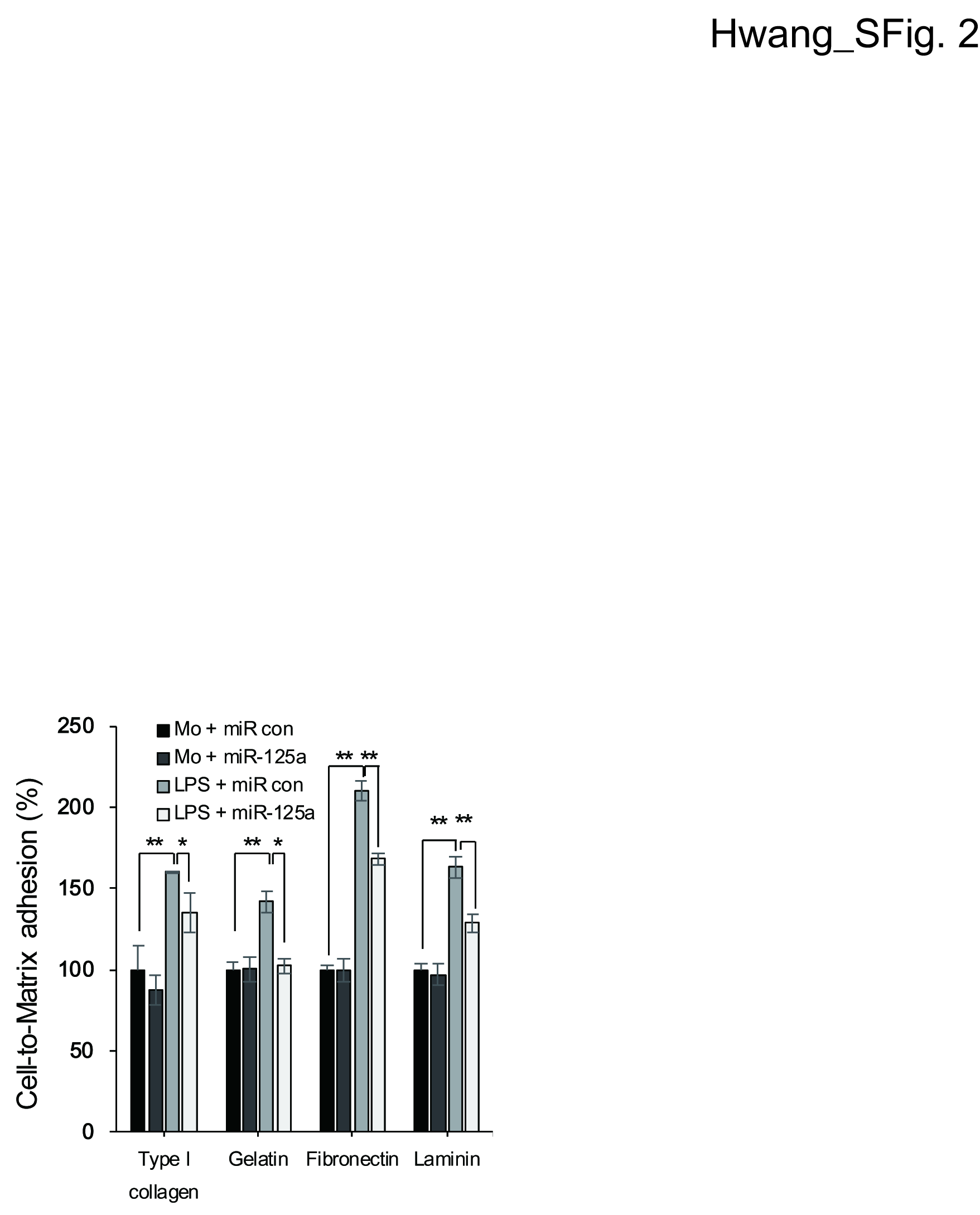

Supplement: Supplementary file 5 — Supplementary Figure S2. [file 41418_2021_911_MOESM5_ESM.tif]

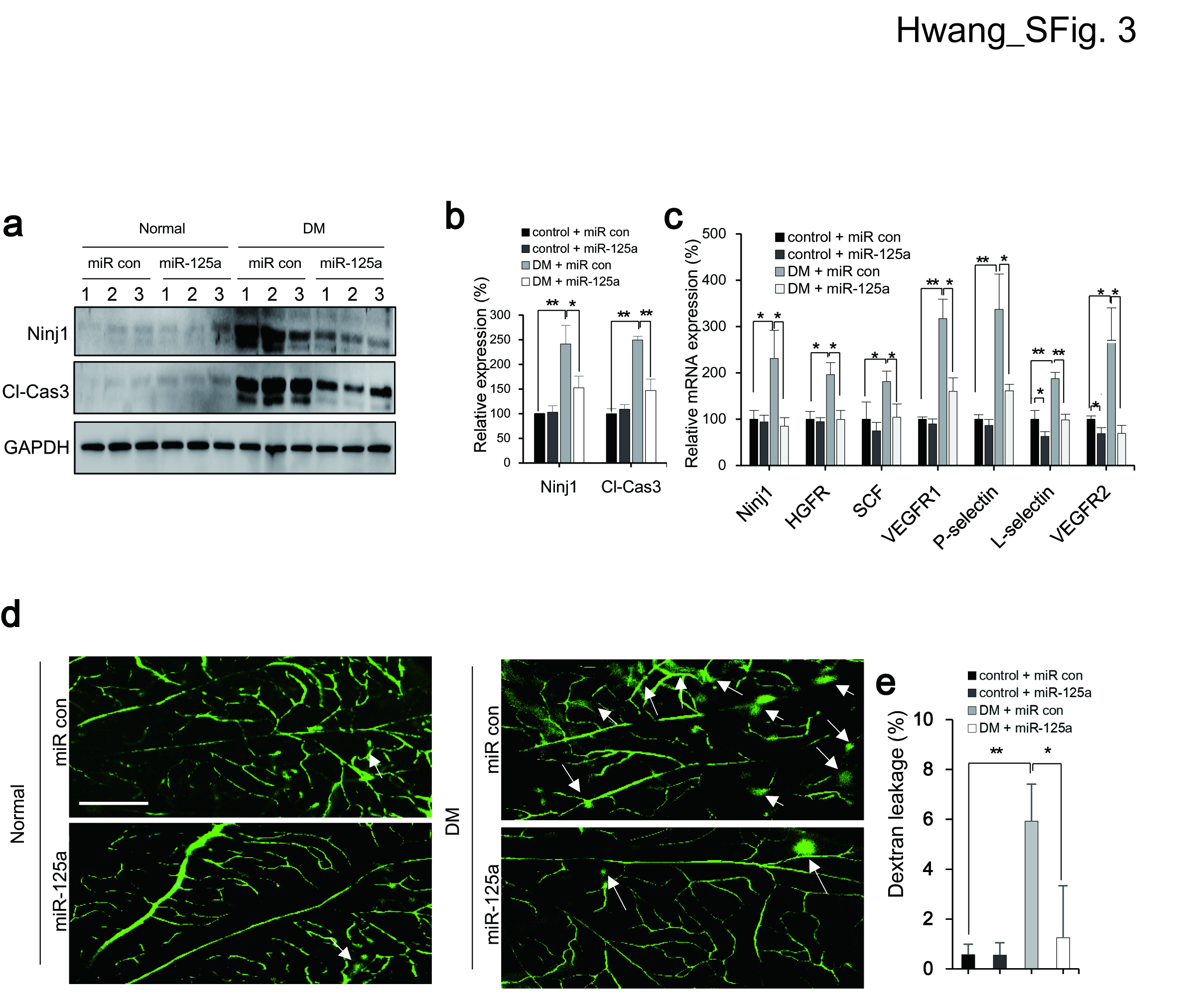

Supplement: Supplementary file 6 — Supplementary Figure S3. [file 41418_2021_911_MOESM6_ESM.tif]

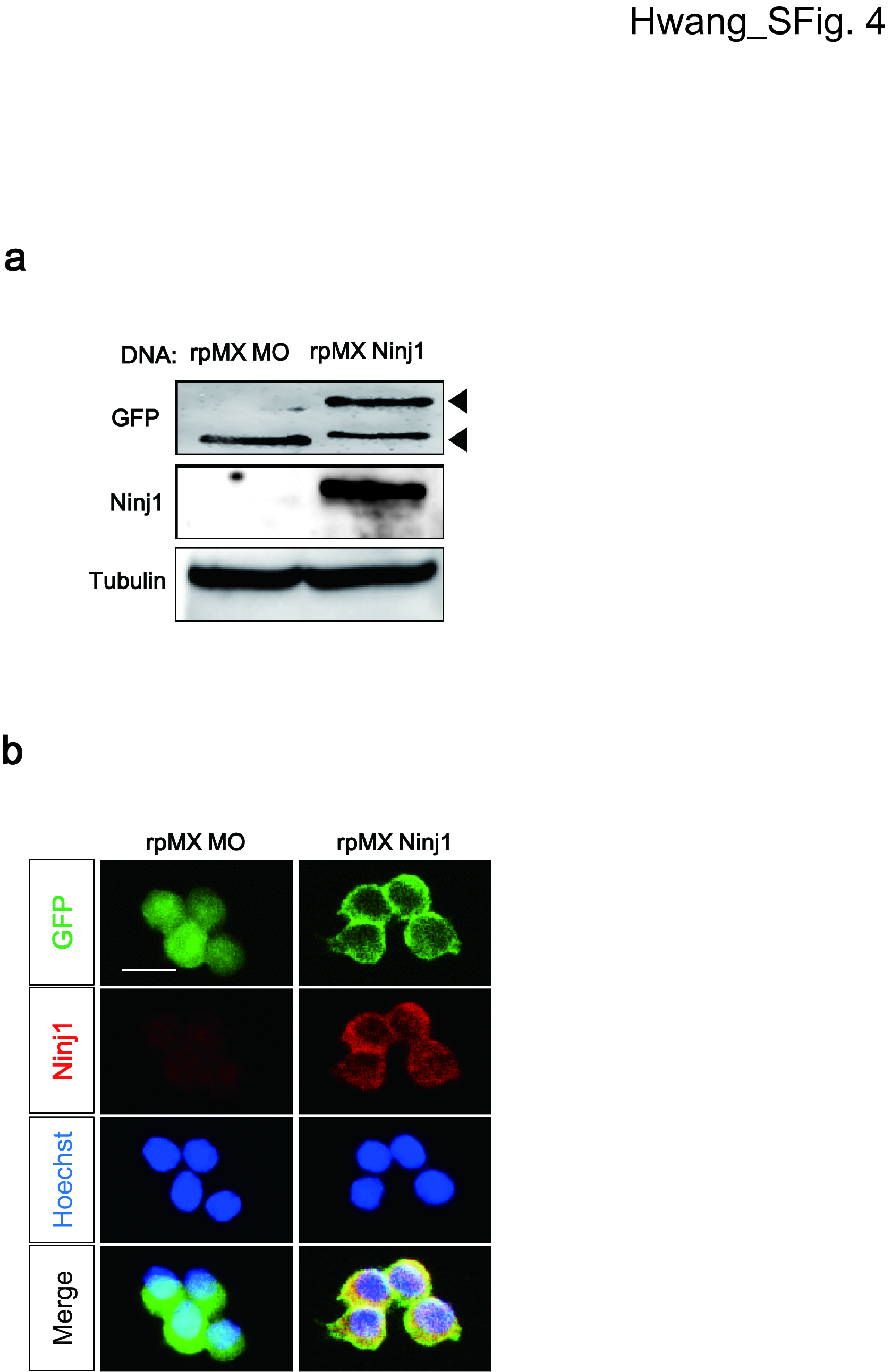

Supplement: Supplementary file 7 — Supplementary Figure S4. [file 41418_2021_911_MOESM7_ESM.tif]
